# Health Professionals’ readiness to implement electronic medical record system at three hospitals in Ethiopia: a cross sectional study

**DOI:** 10.1186/s12911-014-0115-5

**Published:** 2014-12-12

**Authors:** Senafekesh Biruk, Tesfahun Yilma, Mulusew Andualem, Binyam Tilahun

**Affiliations:** Department of Health informatics, Teda Health Science College, Gondar, Ethiopia; Department of Health informatics, University of Gondar, Gondar, Ethiopia; Institute of Medical Informatics, University of Münster, Münster, Germany

**Keywords:** Electronic medical record system (EMR), EMR system, EMR implementation, EMR readiness, EMR utilization, Associated factors of EMR, EMR adoption, Ethiopia

## Abstract

**Background:**

Electronic medical record systems are being implemented in many countries to support healthcare services. However, its adoption rate remains low, especially in developing countries due to technological, financial, and organizational factors. There is lack of solid evidence and empirical research regarding the pre implementation readiness of healthcare providers. The aim of this study is to assess health professionals’ readiness and to identify factors that affect the acceptance and use of electronic medical recording system in the pre implementation phase at hospitals of North Gondar Zone, Ethiopia.

**Methods:**

An institution based cross-sectional quantitative study was conducted on 606 study participants from January to July 2013 at 3 hospitals in northwest Ethiopia. A pretested self-administered questionnaire was used to collect the required data. The data were entered using the Epi-Info version 3.5.1 software and analyzed using SPSS version 16 software. Descriptive statistics, bi-variate, and multi-variate logistic regression analyses were used to describe the study objectives and assess the determinants of health professionals’ readiness for the system. Odds ratio at 95% CI was used to describe the association between the study and the outcome variables.

**Results:**

Out of 606 study participants only 328 (54.1%) were found ready to use the electronic medical recording system according to our criteria assessment. The majority of the study participants, 432 (71.3%) and 331(54.6%) had good knowledge and attitude for EMR system, respectively. Gender (AOR = 1.87, 95% CI: [1.26, 2.78]), attitude (AOR = 1.56, 95% CI: [1.03, 2.49]), knowledge (AOR = 2.12, 95% CI: [1.32, 3.56]), and computer literacy (AOR =1.64, 95% CI: [0.99, 2.68]) were significantly associated with the readiness for EMR system.

**Conclusions:**

In this study, the overall health professionals’ readiness for electronic medical record system and utilization was 54.1% and 46.5%, respectively. Gender, knowledge, attitude, and computer related skills were the determinants of the presence of a relatively low readiness and utilization of the system. Increasing awareness, knowledge, and skills of healthcare professionals on EMR system before system implementation is necessary to increase its adoption.

## Background

With the advance of information communication technologies (ICTs) in the last 20 years, different systems are being implemented in healthcare organizations to improve healthcare services with better data management, communication, and decision making. Out of these, implementing the Electronic Medical Record (EMR) System is the priority agenda not only in developed countries but also in many developing countries [1]. The national e-health strategy toolkit developed by WHO and ITU [2] defines EMR as “a computerized medical record used to capture, store, and share information among healthcare providers in an organization, supporting the delivery of health services to patients”. EMR is perceived as a way to improve healthcare quality through improving work flow, reducing medical errors, minimizing cost and treatment time, increasing revenue, improving patient care by creating a better linkage to all care givers, reducing the need for file space, supplies, and workers for the retrieval and filing of medical records [3-5].

Even though there is a high expectation for and interest in EMR as great potential for improving quality, continuity, safety, and efficiency in healthcare worldwide, the overall adoption rate is relatively low [6-8]. More than 50.0% of the EMRs either fail or fail to be properly utilized in the world [7,9].

The key causes for the low EMR adoption are not only the challenges that follow implementation but also the lack of pre implementation activities, like resource and organizational readiness [10-12] which can facilitate the success of the system implementation. However, only a few studies have been conducted or reported on the readiness of health professionals prior to the actual system implementation. For developing countries, the World Health Organization (WHO) had developed a manual [13] which outlines the preliminary necessary issues, such as organizational, technical, infrastructural, and health professionals readiness for a new work practice of EMR. Nevertheless, most pre implementation assessments failed to include the health professionals’ knowledge and attitude (readiness) which might be a determining factor for the success of the implemented EMR System.

The electronic medical record readiness assessment, as part of a pre implementation assessment is considered essential and must include the human factors [14]. Readiness assessment aims to evaluate the preparedness of each organizational component for a new system implementation [11]. Therefore, identifying areas and requirements before the implementation will help to identify the areas of focus which need to be done during implementation.

One major barrier to successfully implement an EMR system reported by many studies [15-19] is whether clinicians accept the new system and the potential disruptions and changes that follow. It has been suggested that health workers perceived EMR as interfering with clinical workflow, reducing productivity, and introducing disruptive changes to the workplace. This tendency is much more serious in developing countries where computer anxiety is very high [20].

In the Fourth Health Sector Development Plan, the Ethiopian Government had insisted that EMR must be implemented at the major hospitals. The Ministry of Health in collaboration with Tulane University Technical Assistance Project in Ethiopia (TUTAPE) developed a comprehensive EMR system called SmartCare. So far, the system has been deployed in more than eleven hospitals and clinics in Diredawa, Bahirdar, Harar, and Addis Ababa city administrations of Ethiopia as a pilot, and the Ministry of Health planned to scale it up to other hospitals and regions [21].

In its pilot implementation phase, user resistance was reported to be the primary hindering factor to its successful adaptation. Therefore, it is essential to assess the level of readiness of health professionals in front line hospitals and determine the measures to be taken during the implementation of the system in the months ahead. In Ethiopia, no study assessed the readiness and attitude of health professionals for computerization during a pre implementation phase. This study therefore aimed to determine the readiness of health professionals for EMR system implementation, use, and associated developments in hospitals in northwest Ethiopia, which are only months away from the implementation of EMR system. The findings of this study will serve North Gondar Zone, the Amhara Regional Health Bureau, the hospitals under study, health professionals, and hospitals in other similar settings as important evidence to plan and make interventions.

## Methods

An institutional based cross-sectional quantitative study was carried out from January to July 2013 at three hospitals in the northern part of Ethiopia. The study was conducted in North Gondar Zone, northwest Ethiopia. Within the zone, there are three hospitals, named Metema, Debark, and University of Gondar Hospitals. Metema and Debark Hospitals are district hospitals and University of Gondar Hospital is a specialized referral hospital that serves a catchment area of more than 2 million people. In the three hospitals, there is EMR system called the SmartCare Software operating only in the patient registration department with a plan of expanding it to other main clinical departments. Currently, the system is used only to register patients’ socio demographic and some clinical data at the triage level. It also allows users to quickly identify and locate patient history cards. Provision for the use of the International Classification of Diseases, version 10 (ICD-10) is included in the EMR system but is utilized only by the department of Antiretroviral Therapy, not by outpatient, pharmacy, and radiology departments. The latter two are not linked and computerized. In the ART Department, there is a special purpose software designed to register ART related data. Unlike Debark and Metema Hospitals, the Laboratory Department of University of Gondar Hospital is automated with laboratory software used to register laboratory requests and results.

No interoperability and standards are currently used in the three hospitals. Individual identification is being given sequentially while there are no facility codes as the facilities are identified by names.

All the 606 health professionals who were working in the three hospitals (496 at University of Gondar Hospital, 52 at Debark Hospital, and 58 at Metema Hospital) were included in the study for higher precision and accuracy.

Core Readiness is defined as the realization of needs and expressed dissatisfaction with the current way of working while Engagement Readiness is defined as active willingness and participation of people for EMR implementation. We defined Overall readiness as the intersection of core readiness and engagement readiness. That is, health professional is said to have overall readiness he or she must have both engagement readiness and core readiness [22]. We determined core readiness by the different difficulties they face in their current working practice. If health professionals mark at least two problems, like inefficient documentation of patient records, dissatisfaction with the completeness and accuracy of patient data, as well as difficulty in sharing patient records in the questionnaire we assumed they have core readiness for EMR system. We measured the engagement readiness of health professional in terms of their fear or concern about the potentially negative impacts, their recognition of the benefits of EMR, and their willingness to accept EMR training. Both core and engagement readiness were measured by a set of four questions each, and participants who scored more than the mean were categorized as ready and less than the mean were categorized as not ready. Both Core and engagement readiness help people weigh the advantages and disadvantages of the new system in terms of their personal contexts, assess the risk, and question EMR as a solution. The questions regarding core and engagement readiness were validated and used in different studies of EMR and telehealth projects [23-26].

Based on the above framework, participants were asked to measure their current knowledge, attitude, and current utilization of the EMR system (if any, for example, University of Gondar Hospital has standalone EMR system in the laboratory department) as well as their core and engagement readiness levels. Attitude was defined as user feelings about the EMR system if implemented, and knowledge was defined as levels of existing knowhow about the EMR systems. Both attitude and knowledge were measured by a set of four questions each, and participants who obtained more than the mean score were categorized as good and those who scored less than the mean were categorized as poor. Professionals who answered more than 50% of the knowledge, attitude, and readiness questions were classified as good and those who scored less than 50% were classified as poor.

A structured self-administered questionnaire was prepared based on the WHO EMR readiness evaluation framework and additional literature [10-12]. The questionnaire comprised of socio demographic, behavioral (knowledge and attitude), technical, and organizational variables. It was prepared in English, translated into Amharic (local language), and then back to English to check its consistency. The tool was pretested on a group of 30 health professionals who did not belong to the study hospitals. The necessary corrections were made on the questionnaire based on the pretest results.

Six data collectors and two supervisors participated in the data collection process. One day training was given to the data collectors and supervisors on the objective of the study and data collection procedures. The principal investigator and supervisors did a daily supportive supervision on data collectors. Data back-up activities, like storing data at different places and putting data in different formats (hard and soft copies) were performed to prevent data loss.

Ethical clearance for this study was obtained from the Institutional Ethical Review Board of the University of Gondar, Ethiopia. Written consents were taken from the administrations of the respective hospitals. Informed verbal consent was also obtained from individual study participants after an explanation of the study objectives and data confidentiality issues.

The principal investigator and supervisors did frequent data editing manually. Epi Info version 3.5.1 and SPSS version16 were used to enter and analyze data, respectively. Manually edited data were entered into the computer using the data entry template created on Epi Info version 3.5.1 for further analysis. Then the data were exported and analyzed using SPSS version 16. Descriptive statistics was computed to describe the study objectives in terms of appropriate variables. Binary and multi-vitiate logistic regression analysis were carried out to identify the most important variables which could determine EMR readiness and use of health professionals in the study area. Variables with a p-value of ≤ 0.2 on binary logistic regression analysis were entered and further computed on the multi-variate logistic regression model. Associations between the study and outcome variables were described using odds ratio at 95% CI.

## Results

### Socio demographic characteristics

A total of 606 health professionals participated in the study. Of the respondents, 496 (81.8%), were from the University of Gondar Hospital. The mean standard deviation age of the respondents was 26.73 ± 3.99 years with a range of 21 to 50 years. The majority (83.8%) of the study participants were in the age range of 21-29 years (Table 1).

**Table 1:** **Socio-demographic characteristics of health professionals in North Gondar Zone, northwest Ethiopia, 2013**

**Variables**	**Category**	**Number**	**Percent (%)**
Gender	Male	377	62.2
	Female	229	37.8
Age	21-29	508	83.8
	30-34	67	11.1
	35+	31	5.1
Educational status	Diploma and below	167	27.6
	Degree and above	439	72.4
Profession	General practitioner	36	5.9
	Health officer	15	2.5
	Midwifery	43	7.1
	Radiology	16	2.6
	Optometry	11	1.8
	Pharmacy	46	7.6
	Laboratory	84	13.9
	Nurse	319	52.6
	Physiotherapy	17	2.8
	Anesthesia	11	1.8
	Environmental health	8	13
Income	1000-1500	101	16.7
	1600-2500	236	38.9
	2600-3500	225	37.1
	>3600	44	7.3
Length of stay	< 6 months	107	17.7
	6-12 months	149	24.6
	13-18 months	125	20.6
	19-24 months	64	10.6
	>24 months	161	26.6
Computer literate	Yes	356	58.7
	No	250	41.3
Computer access at work place	Yes	473	78
	No	133	22
Computer access at home	Yes	204	36.7
	No	402	63.3

Regarding educational status, 439 (72.4%), of the health professionals had a bachelor’s degree and above, while 167 (27.6%) had diploma and certificate. One hundred fifty-five (25.6%) of the study participants had been working in the study area for less than two years. 187 (30.9%), 133 (21.9%), and 131 (21.6%) of the respondents had working experiences of two to three years, three to five years, and more than five years, respectively. Out of all participants, 319 (52.6%), were nurses, 84 (13.9%) laboratory technicians, 46 (7.6%) pharmacists, and 43 (7.1%) were midwives by profession. More than half, 356 (58.7%), of the respondents were computer literate and 204 (36.7%) had computer access at home.

### Readiness and utilization of health professionals towards EMRs

**Figure 1:**
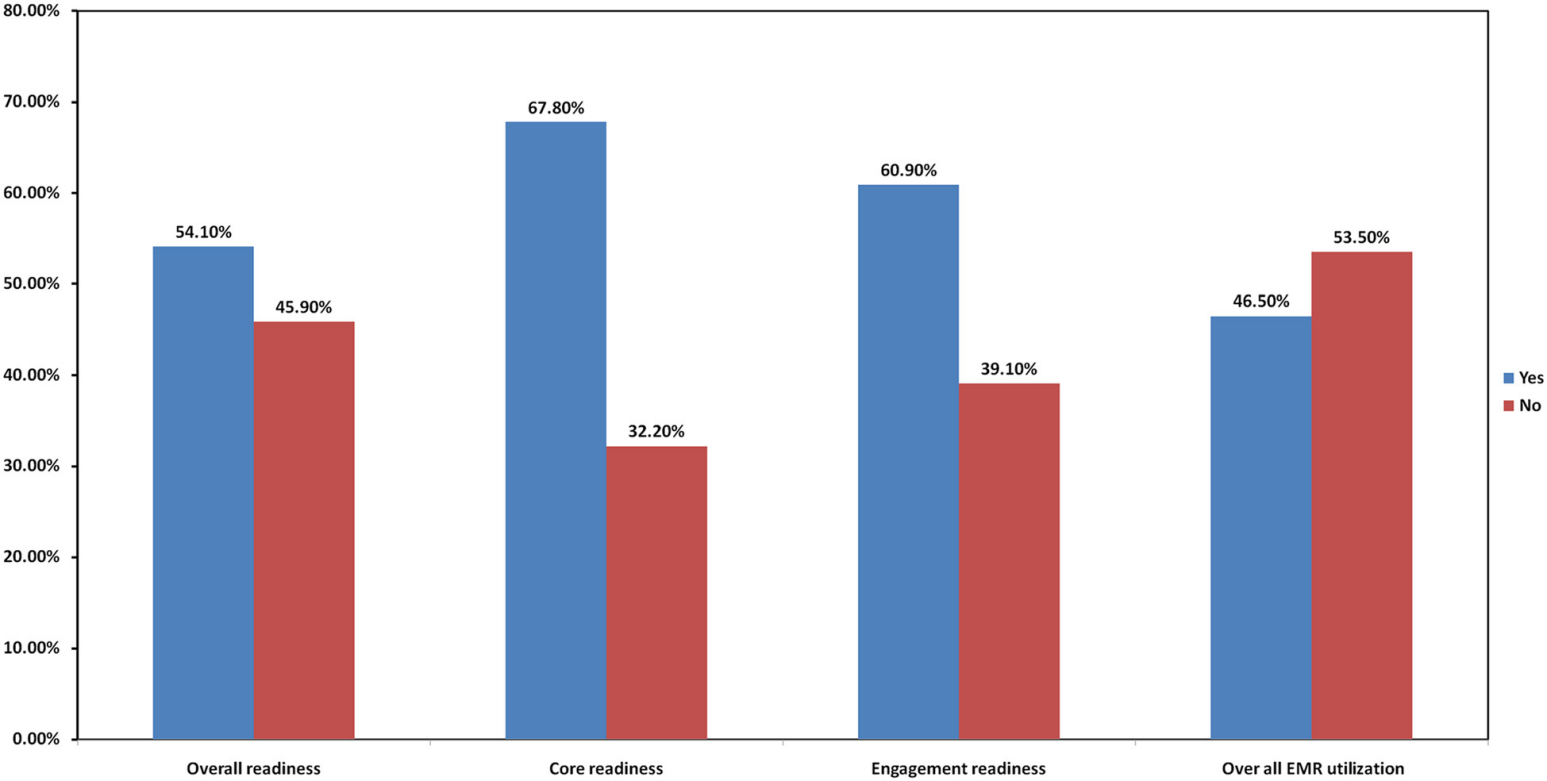
**Health professionals’ Readiness for EMR system in North Gondar Zone, northwest Ethiopia, 2013.**

From the total participants, 328 (54.1%) had overall readiness towards EMR with 411(67.8%) demonstrating core readiness and 369(60.9%) demonstrating engagement readiness towards EMR. The overall existing EMR utilization in the study area was 282 (46.5%) (Figure 1).

### Knowledge and attitude of health professionals for EMR

The majority of the study participants, 432 (71.3%), and 331(54.6%) had good knowledge of and attitude to EMR system, respectively. About 582(96.0%) of the respondents did not have any knowhow about EMR system. The majority, 566 (93.4%), 544 (89.8%), and 544 (89.8%) of the participants said that the hospitals had no good infrastructure; or computers and related technologies; and that they did not have computer related skills, respectively. 540 (89.1%) of the health professionals knew that EMR had great importance for the healthcare system (Table 2).

**Table 2:** **Knowledge and attitude of health professionals toward EMR system in North Gondar Zone, northwest Ethiopia, 2013**

**Variables**	**Category**	**Number**	**Percent (%)**
Knowledge	Yes	432	71.3
	No	174	28.7
Attitude	Yes	331	54.6
	No	275	45.4
Previous knowhow on EMR	Yes	24	4
	No	582	96
Presence of good infrastructure	Yes	40	6.6
	No	566	93.4
Presence of computer & other technologies	Yes	62	10.2
	No	544	89.8
Computer related skills	Yes	62	10.2
	No	544	89.8
EMR has importance:	Yes	540	89.1
	No	33	5.4
	Don’t know	33	5.4
Improve service quality	Yes	404	66.7
	No	202	33.3
Save money & time	Yes	377	62.2
	No	229	37.8
Increase patient satisfaction	Yes	331	54.6
	No	275	45.4
Easy data access	Yes	326	53.8
	No	280	46.2

### Factors associated with health professionals’ readiness for EMR system

In this study, variables like gender, age, good knowledge, having good attitude, willingness to implement EMR system, computer literacy, computer related skills, availability of computers in working place and home, past information technology experience, training access, complexity of system, and level of experience were positively associated with the readiness and use of EMRs by health professionals.

Male health professionals were 1.87 times more likely to be ready for EMR system than female health professionals (AOR = 1.87, 95% CI: [1.26, 2.78]). Similarly, study participants who had good knowledge on EMR system were about 2.12 times more likely to get ready for EMR system as compared to those health professionals with poor knowledge (AOR = 1.87, 95% CI: [1.32, 3.52]). Health professionals who were willing to implement EMR system were 2.63 times more likely to be ready for EMR system than their counterparts (AOR = 2.63, 95% CI: [1.45, 4.78]). Respondents with previous IT experience were 1.69 [1.12, 2.34] times more ready to use EMR system compared with their counter parts (Table 3).

**Table 3:** **Bi-variate and Multi-variate analysis of factors associated with readiness of health professionals for EMR in northwest Ethiopia, 2013**

**Variables**	**Category**	**Readiness**		**Crude OR (95%CI)**	**Adjusted OR (95%CI)**
		**Ready**	**Not ready**		
Gender	Male	226(59.9%)	151(40.1%)	1.86(1.33,2.59)	1.87(1.26,2.78)
	Female	102(44.5%)	127(55.5%)	1	1
Computer literate	Yes	232(65.2%)	124(34.8%)	3.00(2.156,4.19)	1.64(1.99,2.68)
	No	96(38.4%)	154(61.6%)	1	1
Computer skill	Yes	276(62%)	169(38%)	3.42(2.34,5.02)	2.55(1.62,3.76)
	No	52(32.3%)	109(67.7%)	1	1
Presence of Computer in workplace	Yes	281(59.4%)	192(40.6%)	2.68(1.79,3.99)	1.78(1.15,2.77)
	No	47(35.3%)	86(64.7%)	1	1
Presence of Computer at home	Yes	141(69.1%)	63(30.9%)	2.57(1.80, 9.67)	1.71(1.15,2.54)
	No	187(46.5%)	215(53.5%)	1	1
Willingness to implementation of EMR	Yes	302(61.3%)	191(38.7%)	5.67(3.48,9.23)	2.63(1.45,4.78)
	No	26(23%)	87(77%)	1	1
EME Knowledge	Yes	279(64.6%)	153(35.4%)	4.65(3.17,6.83)	2.12(,1.32,3.56)
	No	49(28.2%)	125(71.8%)	1	1
EMR Attitude	Yes	167(50.5%)	164(49.5%)	2.79(1.94, 4.02)	1.56(1.03,2.49)
	No	161(58.5%)	114(41.5%)	1	1
Presence of organizational problems	Yes	236(57.8%)	172(42.2%)	1.97(1.39, 2.76 )	
	No	92(46.5%)	106(53.5%)	1	
Presence of technical problems	Yes	214(63.5%)	123(36.55)	0.42(0.30,0.59 )	0. 32(0.12,0.42)
	No	114(42.4%)	155(57.6%)	1	1
Presence of financial problems	Yes	275(56.6%)	211(43.4%)	1.65 (1.10, 2.46)	
	No	53(44.2%)	67(55.8%)	1	
IT experience	Yes	191(61%)	122(39%)	1.78(1.29, 2.46)	1.69(1.12, 2.34)
	No	137(46.8%)	156(53.2%)	1	1

## Discussion

The present study assessed the readiness of health professionals in three hospitals that were in the frontline to implement the EMR system in the coming years. In our assessment, the overall readiness of those health professionals for an EMR system was 54.1% (with 67.8% core and 60.9% engagement readiness) and can be regarded as low. As health professionals are the main actors in the adaptation and sustainability of the system, interventions are needed in building attitude on the EMR systems. A similar study conducted in Afghanistan (Kabul and Bamyan) on health needs and ehealth readiness assessments of healthcare organizations also found that core readiness for EMR system was 66.7% in Byaman. The result is similar to that of the current study (67.8%) but lower than that in Kabul (35.5%) [27].

The result revealed that health professionals aged 30-34 years were 52% less likely to be ready for EMR system than younger health professionals (AOR = 0.48, (95% CI): 0.24, 0.94). A study conducted in Kuwait also indicated that younger health professionals had better readiness for EMR system [28]. This may be due to the fact that younger people natural tend to have more motive, interest, and readiness to accept new technology developments than aged people.

This study showed that health professionals who were willing to accept the implementation of EMR system were 2.63 times more likely to be ready for EMR system (AOR = 2.63, (95% CI): 1.45, 4.78). Those who had good attitude were 1.56 times more likely to have readiness for EMR system as compared to those health professionals who had poor attitude (AOR = 1.56, (95% CI): 1.03, 2.49). This may be so because favoring the implementation of EMR system may influence the readiness for the system. As discussed in many other studies [6,9,29] health professionals who have good attitude about computerization are more likely to adapt the system. This is also a good explanation for the need to create awareness about EMR system before implementation in order to engage professionals during the system implementation so that they will have good attitude and develop their readiness for a better adaptation of the system.

The present study also identified that health professionals who had good knowledge on EMR system were about 2.12 times more likely to be ready for EMR system as compared to health professionals with poor knowledge (AOR = 2.12, (95% CI): 1.32, 3.56). This may be due to the fact that health professionals who have good knowledge may have the tendency to accept the advantage of technology and to be likely to be ready for EMR system.

The present study also revealed that health professionals who were computer literate, current computer users, and had computers at work place were about 1.64 (AOR = 1.64, (95% CI): 0.99, 2.68), 2.55 (AOR = (2.55, (95% CI):1.62, 3.76), and 1.78 (AOR = 1.78, (95% CI):1.15, 2.77) times more likely to be ready than their counter parts, respectively. A similar study conducted on implementing EMR system in developing countries showed that poor computer skills of healthcare professionals was highly related with poor readiness for EMR system [9,14,25,30,31]. The possible reason for this could be that being computer literate and the availability of computer had a direct influence on health professionals’ views on computer based system use.

This study also showed that health professionals who believed that there was good technical infrastructure for EMR system were 1.78 (AOR = 1.78, (95% CI): 1.01, 3.17) times more likely to be ready for EMR system. This result is comparable to previous systematic reviews conducted on the barriers to the acceptance of EMR system [17]. This particular result is good evidence of the need to discus and promotes EMRs to health professionals before implementation. Just showing the server and the computer infrastructure available for the implementation, can build the confidence of professionals in the success of the implemented system.

From this study, we found out that knowledge and readiness for a computer-based system was relatively low. This can be attributed to the overall technological culture of the society where computer and ICT use is very low and needs more awareness creation before the actual EMR implementation.

## Conclusion

The overall readiness of health professional for EMR system in the study areas was 54.1%. The core and engagement readiness of the study participants were 67.8% and 60.9%, respectively.

Gender, age, computer literacy, knowledge, good attitude, computer related skills, availability of computer in working place and home, past information technology experience, training access, and complexity of the system were the most determinant factors for readiness of health care professionals for EMR system. In addition to training on how to use EMR system and purchasing computers, EMR system implementers need prior discussion and promotion of EMR systems to the potential primary users to increase its adaption and success rate.
